# Interactions between parenting styles, child anxiety, and oppositionality in selective mutism

**DOI:** 10.1007/s00787-024-02484-w

**Published:** 2024-06-04

**Authors:** Ortal Slobodin, Maayan Shorer, Gilor Friedman Zeltzer, Silvana Fennig

**Affiliations:** 1 School of Education, Ben-Gurion University, 84105 Beer-Sheva, Israel; 2 Department of Clinical Psychology and the Lior Tzfaty Mental Pain Center, Ruppin Academic Center, Emek Heffer, Israel; 3 Schneider Children’s Medical Center of Israel, Department of Psychological Medicine, Petah Tikva, Israel; 4 Department of Clinical Psychology, Ruppin Academic Center, Emek Heffer, Israel

**Keywords:** Anxiety, Oppositionality, Parenting style, Selective mutism

## Abstract

Selective mutism (SM) is a poorly understood condition, and debate continues regarding its etiology and classification. Research suggests that a genetic vulnerability may play a role in the development of the disorder which may be compounded by anxious and over-protective parenting. While previous studies supported the role of parenting styles in the development of SM, most of them examined child and parent factors in isolation. The current study examined how the interactions between child internalizing and externalizing behaviors (anxiety and oppositionality, respectively) and parenting styles (authoritative, permissive, and authoritarian) are associated with SM diagnosis. The study included 285 children aged 3–7 years (57.2% females), and their parents (66 children with SM and 219 typically developed children). Parents completed questionnaires about child social anxiety, oppositional behavior, SM severity, and their parenting style. Results showed that parents of children with SM reported lower levels of authoritative practices than those of typically developed children. We also found that child social anxiety and oppositionality moderated the effects of authoritative and authoritarian parenting practices on SM diagnosis. Our results suggest that child anxiety and oppositionality may explain the different susceptibility of children to adaptive and maladaptive parenting styles.

## Introduction

Selective mutism (SM) is a psychiatric condition characterized by a consistent failure to speak in certain social situations, such as school or work, despite the presence of normal speech in other situations [4]. Current views of children's anxiety disorders have highlighted the importance of understanding child anxiety as being multi-determined with genetic, temperamental, and environmental factors, including parental psychopathology, parenting practices, school environment, and sociocultural values [20, 37]. Such an ecological perspective provides an appropriate conceptual framework to understand the complexity and heterogeneity of SM because it situates children's mental health problems within a rich theoretical framework that relates environmental factors with child-related variables [48]. The current study aimed to examine how the interactions between children’s internalizing and externalizing behaviors (anxiety and oppositionality, respectively) and parenting styles (authoritative, permissive, and authoritarian) are associated with SM diagnosis. This investigation may shed light on the mechanisms of the disorder and may contribute to the development of future interventions [11].

## Selective mutism

SM is more common than previously considered, with an estimated prevalence rate of 0.7 and 2% [38]. The disorder is more prevalent in girls than in boys with a gender ratio of 1.5:1 to 2.1:1 [27, 28]. Symptoms of SM are recognized as early as 2 years, but the disorder is often diagnosed only when children enter school [49]. SM is a poorly understood condition, and its etiology and classification are still debated [26]. However, research points to various individual, familial, and contextual risk factors.

At the child's level, etiological explanations include heightened anxiety, particularly social anxiety disorder [12], emotional dysregulation [33], language/communication deficits (Cohan et al., 2008), social skills deficits [10, 35], and behavioral inhibition, especially in unfamiliar situations [17]. At the family level, SM was associated with parental psychiatric disorders Koskela et al. [25] and harsh parenting behaviors [52]. Together with the above etiological factors, SM was associated with socio-cultural variables, including immigration background [14] bilingualism, and socioeconomic status [46, 47]. As the current study focuses on the interaction between parent and child SM risk factors, we will now elaborate on the relationship between parenting styles and SM.

## Parenting styles and SM

Previous studies have pointed to the role of parenting style in the development and maintenance of SM [13, 52]. Baumrind’s [5] conceptualization of parenting style included parents’ attitudes, perceptions, values, and behaviors. She described three parenting styles that are derived from the parents’ balance of emotional warmth/responsiveness and their level of control/demand when interacting with their children. Authoritarian parents attempt to shape, control, and evaluate the behavior and attitudes of the child in accordance with a set of standards of conduct. Permissive parents attempt to behave in a non-punitive, affirmative, and accepting (i.e., warmhearted, affectionate, concerned, Önder and Gülay, 2007) manner towards child impulses, desires, and actions but make only a few behavioral demands. Authoritative parents monitor and guide their child’s activities but in a focused, rational, and empathic manner. Despite being subjected to cultural variation, previous research has suggested that parenting styles that balance between warmth and control (e.g., authoritative) are associated with better child physical and mental health outcomes compared to styles focused on only one of these constructs (e.g., permissive, authoritarian) [23, 32].

To date, studies examining the association between parenting styles and SM are scarce and inconclusive. Yeganeh et al. [52] who examined the relationship between SM, social phobia, oppositionality, and parenting styles in children aged 7–15 years found that children with social phobia and children with SM reported significantly lower parental warmth and acceptance than their typically developed peers. In the same vein, Edison et al. [13] found that the parents (mostly mothers) of children with SM were more controlling and over-protective than the parents of control children or children with other anxiety disorders (i.e., specific phobia, generalized anxiety disorder, panic disorder, social phobia, separation anxiety disorder, obsessive–compulsive disorder, posttraumatic stress disorder).

The authors suggested that controlling parenting, characterized by lower autonomy granting and an increased proportion of high-power remarks (e.g., commands) that demand a response on the part of the child may contribute to the development of SM by reinforcing child behavioral inhibition and limiting the opportunities for the child to take a communicative risk [13]. By contrast, Cunningham et al. [10] failed to find differences between children with and without SM in parental self-reports of permissive and coercive discipline. Similarly, Alyanak et al. [3] found no differences between parents of children with and without SM in parental self-reports of over-protective parenting style.

Parenting styles may not only serve as etiological factors for SM but may also play an important role in the maintenance and prognosis of the disorder [3]. Parents of anxious children might engage in accommodation behaviors (e.g., modifying family routines, providing excessive reassurance) to reduce child distress. In the long term, however, such strategies have the potential to maintain anxiety and facilitate further avoidance [31]. Although the relationship between parenting styles and accommodation is an understudied area, there is evidence to suggest that both authoritarian and permissive styles may be consistent with parental accommodating behaviors: authoritarian parents prevent children from gaining opportunities to confront and survive challenges themselves, whereas permissive parents may allow their children to avoid anxiety-provoking situations [2].

To date, studies focusing on the development and maintenance of SM examined child and parent factors in isolation. This approach limits our understanding regarding the interaction between child and parent factors in SM. Research has increasingly considered the combined influence of parenting and child risk factors in the development of children’s behavior problems [44], Wittig and Rodriguez [50], suggesting that any given risk factor or parenting style may not be inherently problematic. Rather, the interaction between parenting and child factors may result in children’s maladaptive outcomes. In the current study, we examined how the interactions between child internalizing and externalizing behaviors (anxiety and oppositionality), and three parenting styles (authoritative, permissive, and authoritarian) are associated with SM diagnosis.

## Anxiety and oppositionality in SM

The DSM-5 [4] defines SM as a type of anxiety disorder. However, a growing amount of evidence suggests that children with SM present quite heterogeneous symptoms (Cohan et al., 2008; [22]). Based on exploratory and confirmatory factor analysis of parent ratings of internalizing and externalizing behavior problems, [11] identified two distinct factors related to anxious and oppositional behaviors in SM: The anxious factor, primarily characterized by social withdrawal and the oppositional, primarily characterized by argumentativeness and rebellion. Scores on the latter factor related positively to measures of aggression and symptoms of an oppositional defiant disorder, and inversely to symptoms of social anxiety disorder. In their later investigation based on a larger sample of children with SM, Diliberto, and Kearney [11] revealed three distinct profiles characterized as (1) moderately anxious, oppositional, and inattentive, (2) highly anxious, and moderately oppositional and inattentive, and (3) mildly to moderately anxious, and mildly oppositional and inattentive. The second profile was the most impaired and associated with increased shyness, social problems, and emotionality.

Overall, these theoretical frameworks of SM have gravitated toward how symptom profiles may reflect operant and other factors to help explain the disorder [34]. For instance, internalizing behaviors, such as shyness, behavioral inhibition, social withdrawal, and overdependence suggest that some children with SM fear negative consequences for speaking and seek to decrease anxiety in social situations [41]. In contrast, externalizing behaviors, such as noncompliance, irritability, and willfulness may suggest that some children with SM avoid the anxiety associated with speaking by acting out behaviors, such as coercing parental acquiescence to demands [21].

## The current study

While previous studies of SM addressed child and parent factors separately, growing evidence suggests that children and parents may affect one another in a reciprocal fashion, affecting the development of children’s behavior problems [44, 50]. The current study sought to address current gaps in the SM literature by focusing on the moderating effect of child anxiety and oppositionality on the relationship between parenting styles and SM.

Based on the abovementioned literature, three hypotheses were examined. First, we hypothesized that children with SM would present higher social anxiety and oppositional levels than children without SM. Second, we hypothesized that parents of children with SM would use more authoritarian and permissive parenting behaviors and less authoritative behaviors than parents of children without SM. Finally, we hypothesized that child anxiety and oppositionality levels would moderate the relationship between parenting styles and SM diagnosis. Specifically, we expected that the authoritarian and permissive parenting style would be associated with an increased likelihood of SM in children with higher levels of anxiety and oppositionality than in children with lower levels of anxiety and oppositionality.

## Method

### Participants

Participants were 285 children aged 3–7 years (mean = 5.11, S.D = 0.77, 57.2% females), and their parents. Of the total sample, 66 children were diagnosed with SM, and 219 were typically developed children. In most cases (93%), the mother was identified as the main-caregiving parent and participated in the study. Most parents (87%) were married, and 30% were first-generation immigrants (mostly from the former Soviet Union). Children, however, were mostly native-born (97%). Parents’ mean levels of education were 15.73 years (S. D = 3.03).

## Procedure

Participants in the SM group were clinic-referred children recruited from an outpatient psychiatric clinic, specialized in SM. Participants in this group were recruited during their first visit to the clinic. Inclusion criteria for the SM group was (1) meeting the DSM-5 [4] criteria for SM, as assessed by a certified psychologist using the Anxiety Disorders Interview Schedule for DSM-IV, Parent Version (ADIS-P; [43]), (2) absence of psychiatric disorder (except anxiety), such as autistic spectrum disorders, psychosis, mental disability, and (3) did not use pharmaceutical treatment for anxiety within 2–6 weeks before participation in the study. Participants in the control group were recruited through different online platforms (e.g., e-mails, social media, and online groups) and convenience sampling. The inclusion criteria for participants in the control group were (1) the parent declared that the child was never diagnosed with SM or any other psychiatric disorder (2) an absence of behavioral or academic problems, based on parents' reports, and (3) The child scored below 2.5 in the reversed total score of the Selective Mutism Questionnaire, which is considered a benchmark value for SM [7, 36].

Participation was voluntary and independent of child treatment. Parents in the control group who had more than one child in the target age range were instructed to focus on one child alone. The parent who self-identified as the main caregiver completed all questionnaires online using Qualtrics software. The study protocol met the Helsinki guidelines for research involving human subjects and was approved by the ethics committee of the Schneider Children Medical Center (# RMC-0210–15). All parents signed written informed consent.

## Measures

Background variables included child age, gender, school, medication, place of birth, number of siblings, parent’s education, family income, and number of spoken languages.

The Anxiety Disorders Interview Schedule for DSM-IV, Parent Version (ADIS-P; [43]) was used to establish the diagnostic status in the SM group. A clinical severity rating of 4 on a 0–8 scale is indicative of a clinically significant disorder and was required for an SM diagnosis.

SM symptoms severity was assessed by the Selective Mutism Questionnaire (SMQ; [7]). The SMQ includes 17 items addressing child speech inhibition in three domains: school (five items), family (five items), and social situations” (seven items). For example, "When appropriate, my child talks to selected peers (his/her friends) at school". Parents are asked to rate the frequency of each item, using a 4-point scale ranging from "3″ (always) to "0″ (never). In this study, the SMQ scoring was reversed so that a higher score indicates more SM symptoms. An SMQ total score was calculated as the average of these 17 items, thus ranging between 0 and 51 with higher scores indicate increased SM severity. In addition, there were three overall interference and distress questions, such as “Overall, how much did not talking interfere with daily living for your child?” In these questions parents are asked to rate each item on a 4-point scale ranging from "1″ (not at all) to "4″ (extremely). Bergman et al. [7] reported internal consistency of Cronbach’s *α* = 0.84. In the current study, Cronbach alpha was 0.86.

Social Anxiety Scale for Children-Revised—parents’ version (SASC-R; [30]) is a 22-item measure assessing social anxiety in children. The parents report their children’s emotions and behaviors on 18 anxiety-related items and four filler items (e.g., “likes to read”). Each item can be answered using a five-point Likert-type scale ranging from "1" (not at all) to "5" (all the time). Scores range between 18 and 90, with higher scores indicating higher levels of social anxiety. The scale is composed of three subscales: Fear of Negative Evaluation (eight items), Social Avoidance and Distress-New Situations (six items), and General Social Avoidance and Distress (four items). The scale has an excellent internal reliability, with Cronbach alpha of 0.92 [29]. Cronbach alpha in the current study was 0.87.

A Child Behavior Checklist (CBCL/6–18 and CBCL/1.5–5; [1]) was used to assess child oppositionality. The CBCL is a widely used measure of child behavioral and emotional functioning and is consisted of eight narrowband syndrome scales including, Aggressive Behavior, Anxious/Depressed, Attention Problems, Rule-Breaking Behavior, Somatic Complaints, Social Problems, Thought Problems, and Withdrawn/Depressed. Parents rate child symptoms on a 3-point scale ranging between 0 (not true), and 2 (very/often true). In the current study, we assessed child oppositionality level using Evans et al. [15] two factor model. This model uses CBCL items to calculate a two-dimension model of oppositionality that is composed of irritability (three items: stubborn/sullen/ irritable, mood, temper) and defiance (three items: argues, disobeys-home, disobeys-school). Previous research [15] has shown that irritability was correlated with anxiety and depressive disorders, defiance was associated with conduct disorder, and both with oppositional defiant disorder. Scores range between 0 and 12, with higher scores indicating higher levels of oppositionality. Evans et al. [15] reported Cronbach alpha values of 70 and 0.73, for defiance and irritability, respectively [15]. In the current study, Cronbach alpha of the total oppositionality score (six items) was 0.83.

Parental Authority Questionnaire (PAQ; [8]) is a 30-item self-report questionnaire with three 10-item scales representing authoritative, authoritarian, and permissive parenting styles. Items are rated on a 5-scale ranging from 1 (strongly disagree) to 5 (strongly agree. Subscale scores range from 10 to 50. Higher scores in a certain subscale indicate stronger affiliation with this type of parenting style. The questionnaire has solid psychometric properties, with Cronbach alphas of 0.74–0.87 [8]. In the current study, the permissive scale had a Cronbach *α* = 0.69, the authoritarian scale had Cronbach *α* = 0.79, and the authoritative scale had Cronbach *α* = 0.72.

## Data analysis

First, independent *t* tests were calculated for examining demographic and clinical differences between the two study groups. Next, Pearson correlations between study variables were calculated. Finally, we examined the two-way and three-way interaction effects between parenting style, child anxiety, and oppositionality on the diagnosis of SM. Conditional process modeling was conducted using PROCESS macro model 2, with child gender and age serving as covariates. The PROCESS [18] defines low, medium, and high levels of the moderator as one S.D. below the mean, the mean, and one S.D. above the mean, respectively.

An alpha level of 0.05 was used in all statistical analyses. Power calculations revealed that for independent *t* test with a power of 0.8 and Cohen's d effect size of 0.5, a minimum of 51 participants in each group is required. Multiple regression with five predictors and a power of 0.8 required a minimum of 91 participants. Data were analyzed using SPSS version 26.

## Results

Table 1 presents differences in demographic and clinical variables between children with and without SM. As seen in the table, children with SM were older than children without SM. Partially supporting the first and second hypotheses, children with SM had higher levels of social anxiety than children without SM and their parents reported fewer authoritative parenting behaviors. However, no differences between the two groups were observed in permissive and authoritarian parenting and child oppositionality.

**Table 1:** Differences in demographic and clinical variables between children with SM and typically developed children

	Children with SM diagnosis M (S.D)	Typically developed children M (S.D)	Group difference
Child’s age	5.32 (0.86)	5.06 (0.73)	*t *(95.28) = −2.96*
Family income	2.87 (0.53)	2.91 (0.44)	*t* (87.99) = 2.01*
SM symptoms	1.79 (0.62)	0.85 (0.40)	*t* (82.44) = −11.70***
Anxiety	67.72 (14.87)	50.31 (10.34)	*t* (86.25) = −8.85***
Oppositionality	10.60 (3.10)	10.26 (2.98)	*t* (253) = −0.77
Authoritative parenting style	37.44 (7.43)	40.45 (4.67)	*t* (67.03) = 2.85**
Permissive parenting style	23.36 (6.26)	23.35 (5.43)	*t* (240) = −0.12
Authoritarian parenting style	23.09 (7.41)	22.76 (5.95)	*t* (240) = −0.34

^*^p < 0.05, **p < 0.01, ***p < 0.001

Table 2 presents the Pearson correlation matrix for the study variables. Analysis revealed that child SM symptom level was positively associated with the levels of social anxiety and oppositionality and negatively with the levels of authoritative parenting style. Social anxiety was positively associated with oppositionality and negatively with the authoritative parenting style. Oppositionality was positively associated with an authoritarian parenting style.

**Table 2:** Pearson correlations between study variables

	Child’s age	SM symptoms	Anxiety	Oppositionality	Authoritative parenting style	Permissive parenting style	Authoritarian parenting style
Child’s age	1	0.20**	0.12*	−0.02	0.04	0.08	−0.06
SM symptoms		1	0.59**	0.19**	−0.26**	0.08	0.06
Anxiety			1	0.21**	−0.22**	0.10	−0.01
Oppositionality				1	−0.04	0.02	0.21**
Authoritative parenting style					1	0.05	−0.01
Permissive parenting style						1	0.13*
Authoritarian parenting style							1

^*^p < 0.05, **p < 0.01, ***p < 0.001

**Fig. 1:**
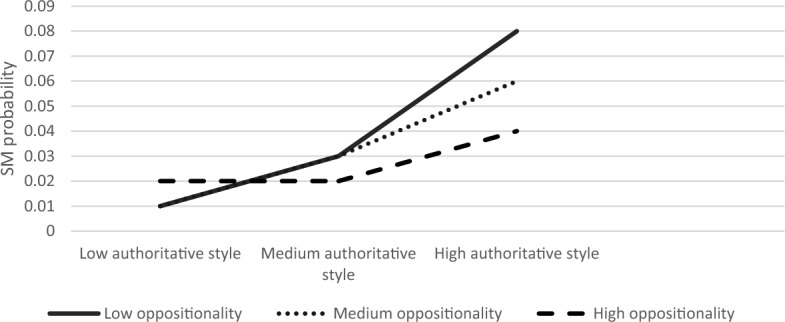
The Interaction Effect between Authoritative Parenting and Child Oppositionality on SM Probability in Low Anxiety Levels. No significant interactions were found

**Fig. 2:**
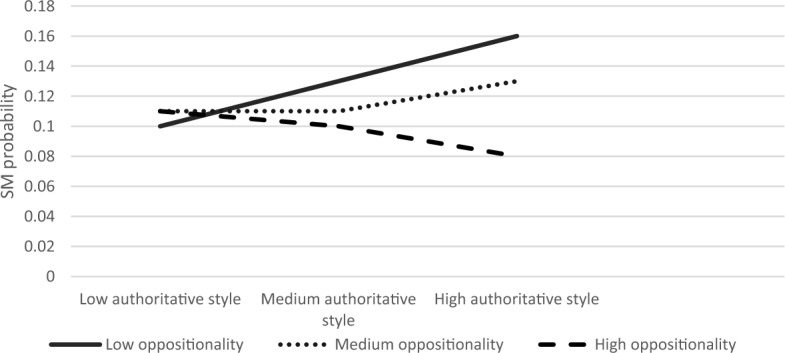
The Interaction Effect between Authoritative Parenting and Child Oppositionality on SM Probability in Medium Anxiety Levels. No significant interactions were found

**Fig. 3:**
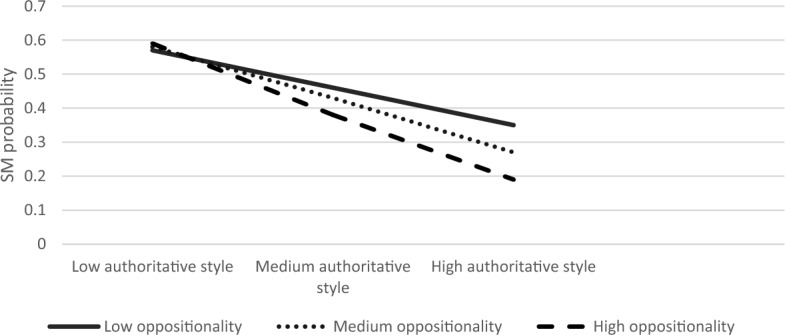
The Interaction Effect between Authoritative Parenting and Child Oppositionality on SM Probability in High Anxiety Levels. Interactions were significant in medium and high levels of oppositionality

Next, we explored the moderating role of child anxiety and oppositionality in the association between parenting style and SM. The first regression examined the association between authoritative parenting style and SM. The overall model was significant Modell LL (7) = 88.37, *p* < 0.001. Results showed that the authoritative parenting style and child anxiety had main effects on the likelihood of SM (B = 0.65, S.E = 0.28, *Z* = 2.34, *p* < 0.05; B = 0.53, S.E = 0.19, *Z* = 2.73 *p* < 0.01, respectively). The interaction effect between authoritative parenting style and child anxiety on SM was significant (B = −0.01, S. E = 0.005, Z = −2.13, *p* < 0.05). Furthermore, the product term of the three-way interaction of authoritative parenting, anxiety, and oppositionality was significant; *χ*^2^ (2) = 7.36, *p* < 0.05. Simple slope analyses revealed that the association between authoritative parenting and SM was significant in children with high anxiety and medium to high oppositionality (high anxiety and medium oppositionality; B = −0.11, S.E = 0.05, Z = −2.14, *p* < 0.05, high anxiety and high oppositionality; B = −0.16, S. E = 0.05, Z = −3.07, *p* < 0.01), but not when high anxiety was coupled with low oppositionality (B = −0.08, S.E = 0.08, Z = −1.07, *p* = 0.28). The results of this model for low, medium, and high levels of anxiety are graphically displayed in Figs. 1, 2, 3, respectively. As seen in Fig. 3, the SM likelihood of children with medium–high oppositionality and high anxiety levels decreased with higher levels of authoritative parenting.

The second regression examined the relationship between permissive parenting style and SM.

**Fig. 4:**
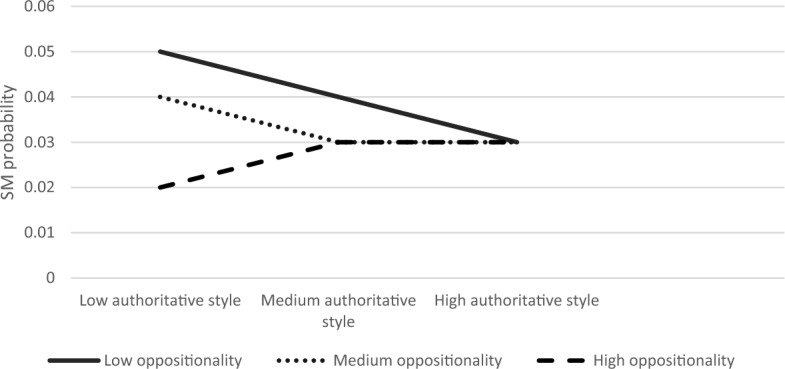
The Interaction Effect between Permissive Parenting and Child Oppositionality on SM Probability in Low Anxiety Levels. No significant interactions were found

**Fig. 5:**
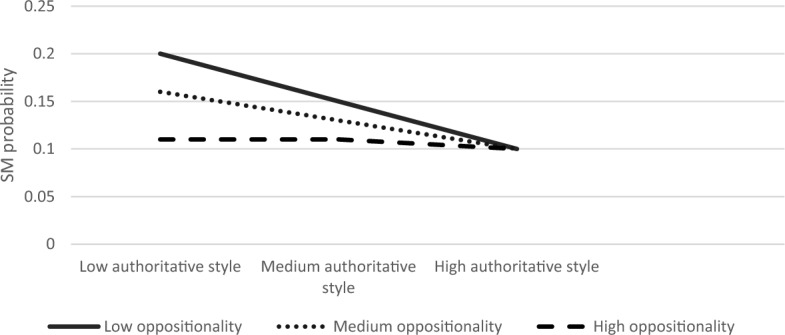
The Interaction Effect between Permissive Parenting and Child Oppositionality on SM Probability in Medium Anxiety Levels. No significant interactions were found

**Fig. 6:**
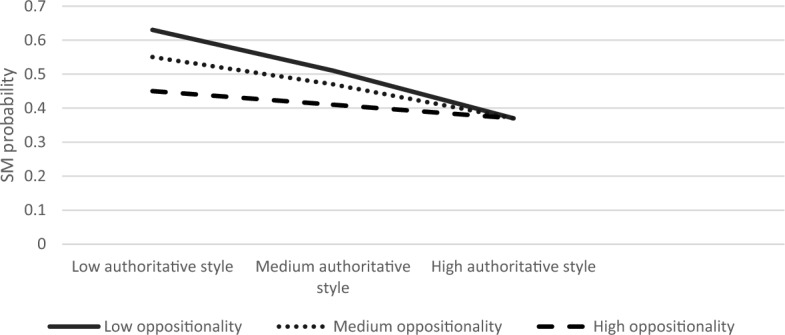
The Interaction Effect between Permissive Parenting and Child Oppositionality on SM Probability in High Anxiety Levels. No significant interactions were found

The overall model was significant ModellLL (7) = 79.65, *p* < 0.001. Results showed that child anxiety was the only predictor of SM (B = 0.18, S.E = 0.08, Z = 2.28, *p* < 0.05). No two-way or three-way interactions were found. Results for each level of anxiety are graphically displayed in Figs. 4, 5, 6.

Finally, we examined the relationship between the authoritarian parenting style and SM.

**Fig. 7:**
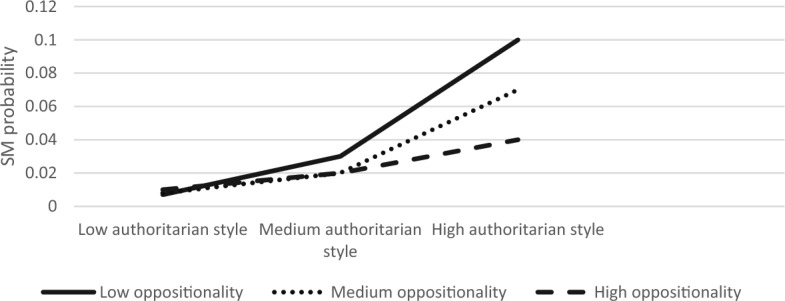
The Interaction Effect between Authoritarian Parenting and Child Oppositionality on SM Probability in Low Anxiety Levels. Interactions were significant in low and medium levels of oppositionality

**Fig. 8:**
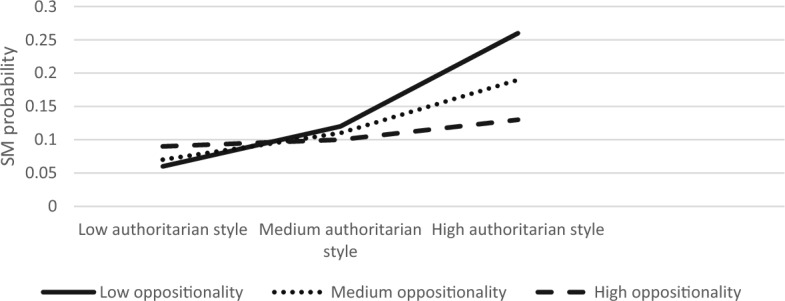
The Interaction Effect between Authoritarian Parenting and Child Oppositionality on SM Probability in Medium Anxiety Levels. Interactions were significant in low and medium levels of oppositionality

**Fig. 9:**
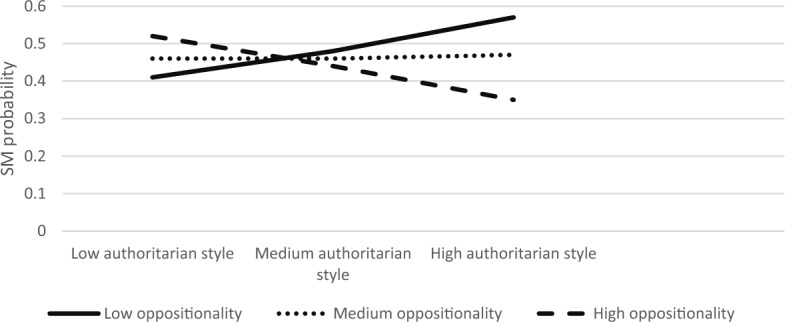
The Interaction Effect between Authoritarian Parenting and Child Oppositionality on SM Probability in High Anxiety Levels. No significant interactions were found

The overall model was significant ModellLL (7) = 84.90, *p* < 0.001. We found that authoritarian parenting and child anxiety had main effects on SM (B = 0.59, S.E = 0.22, Z = 2.66, *p* < 0.01; B = 0.30, S.E = 0.08, Z = 3.77, *p* < 0.001, respectively). The interaction effect between child anxiety and authoritarian parenting style on SM was significant (B = −0.01, S.E = 0.003, Z = −2.33, *p* < 0.05). The three-way interaction between authoritarian style, anxiety, and oppositionality was also significant (*χ*^2^ (2) = 7.54, *p* < 0.05). Simple slope analyses revealed that association between authoritarian parenting and SM was significant in children with low or medium anxiety levels and with low or medium oppositionality (low anxiety, low oppositionality; B = 0.21, S.E = 0.08, Z = 2.65, *p* < 0.01, low anxiety, medium oppositionality; B = 0.17, S.E = 0.06, Z = 2.60, *p* < 0.01, medium anxiety, low oppositionality; B = 0.13, S.E = 0.06, Z = 2.25, *p* < 0.05, medium anxiety, medium oppositionality; B = 0.09, S.E = 0.09, Z = 2.29, *p* < 0.05). Figures 7, 8, 9 present the interactions between authoritarian parenting style and child oppositionality in children with low and medium, and high levels of anxiety, respectively. As seen in Fig. 7, the SM likelihood of children with medium–high oppositionality and low anxiety levels increased with higher levels of authoritarian parenting. Likewise, as seen in Fig. 8, the SM likelihood of children with medium–high oppositionality and medium anxiety levels increased with higher levels of authoritarian parenting.

## Discussion

The current study examined how the interactions between child internalizing and externalizing behaviors (anxiety and oppositionality) and parenting styles (authoritative, permissive, and authoritarian) are associated with SM diagnosis. In line with previous findings [13], Yagenah et al. 2006), our results showed that parents of children with SM showed lower levels of authoritative practices than parents of children without SM. However, no group differences were found in authoritarian and permissive practices, both suggested as etiological factors in SM [40]. Several other studies did not find differences in self-reported maladaptive parenting practices between parents of children with SM and typically developed children [3, 14]. For example, Alyanak et al. [3] found no differences in over-protective parenting (characterized by over-controlling, anxious, and over-demanding parental attitudes) between parents of children with SM and controls. These results may suggest that children with SM may experience lower levels of accepting, guiding, and responsive parenting, but not necessarily higher levels of maladaptive parenting.

Consistent with previous findings documenting parenting-child interaction effects on child development [6, 44], our results suggest that child anxiety and oppositionality may explain the different susceptibility of children to adaptive and maladaptive parenting styles. Prior research showed that children higher on putative sensitivity markers, such as negative emotionality and surgency, are more sensitive to the parenting styles than children lower on these markers [44]. The current study lends further support for this view, suggesting that when child social anxiety is high, especially if coupled with a high oppositionality level, having authoritative parenting would be associated with a lower risk for SM than having a parent with low authoritative practices. That is, children with a combination of anxiety and oppositionality may benefit the most from supporting, responsive, and nurturing parenting that takes the child perspective into account but also provides structure and guidance.

In contrast to our expectations, we found that having an authoritarian parenting style was associated with an increased risk for SM only in children with low or medium levels of anxiety and oppositionality. One possible explanation for these findings is that the positive features of the rearing environment, rather than its negative features, play a key role in the successful adaptation of anxious and oppositional children. For example, [16] found that while exposure to more positive parenting reduces behavior problems in children with difficult temperaments, no moderating effects of temperament were found for negative parenting. Given the high comorbidity between anxiety and SM [12], another possible explanation for our findings is that the contribution of a negative parenting style to the prediction of SM probability was negligible in children with high social anxiety. An alternative interpretation of our findings is related to a ceiling effect on the risk of SM. A ceiling effect might explain why children with high levels of anxiety did not show the same increase in SM probability in response to higher levels of authoritarian parenting as children with lower levels of anxiety.

Notably, child oppositionality did not have a main effect on the risk for SM and did not have two-way interaction effects with different parenting styles. These findings may suggest that the combination of maladaptive parenting style, anxiety, and oppositionality, rather than oppositionality per se, may be considered a risk factor for SM. Probably, SM may develop in those children who react to an anxiety-provoking event in a non-compliant manner, such as a refusal to speak (Keeton 2013). Nevertheless, some researchers suggest that adults may interpret children's avoidance behaviors as controlling or oppositional when they are actually an expression of overwhelming social anxiety [53].

Our findings should be considered under several limitations. First, the current study relied on parental reports of child symptoms and parenting styles. This approach may cause problems of shared method variance, such that the associations obtained between parenting styles and child anxiety, oppositionality, and SM may become artificially inflated. For example, prior work indicates that the effects of negative affect on parenting behavior may differ when parenting is assessed via observation [44]. Multi-informant and observational studies might give more objective information on parent–child interaction [3]. Second, the current study did not distinguish between paternal and maternal parenting practices. Research focusing on parent–child interaction effects on child outcomes suggested that these interactions may differ for fathers and mothers. For example, Wittig & Rodriguez [50] found that urgency moderated the effects of maternal authoritative and paternal permissive parenting styles on infants’ externalizing behaviors and the effects of maternal authoritarian and paternal authoritative parenting styles on infants’ internalizing behaviors. Future studies should focus on the unique contribution of fathers’ and mothers’ parenting practices and their interaction with the development and maintenance of SM. Third, the cross-sectional design of this study limits our ability to draw any conclusions about causality or reciprocal influences between child difficulties and parenting behavior [39]. While maladaptive parenting may contribute to child development problems, this link could also be reversed, so that child difficulties elicit parenting stress, conflict, and dysfunction [24]. A longitudinal approach may add important insight into the developmental trajectories of parent–child interactions and their long-term effects on children outcomes. Fourth, the current study did not consider the influence of SM comorbidities on our findings. SM was associated with a wide variety of comorbidities such as anxiety disorders, enuresis, encopresis, obsessive–compulsive disorder, language and communication problems, and developmental delay [10, 51]. Future studies should address how different comorbidities are related to SM, and how they influence the interaction between child and parent risk factors. Finally, a larger clinical sample balanced with respect to age, socioeconomic background, or aspects of language would be more valid and allow us to generalize our results.

## Conclusions and implications

This study contributes to our understanding of the relationships between parenting styles, children’s anxiety, oppositionality, and SM. The findings add to the existing SM literature by demonstrating that child anxiety and oppositionality moderate the relationship between parenting styles and SM. Our results suggest that positive (i.e., authoritative) parenting may be associated with a reduced risk for SM in children with high levels of anxiety and oppositionality. However, negative parenting was associated with SM only in children who exhibited fewer externalizing and internalizing behaviors. At the theoretical level, the results of this study highlight the importance of developing an ecological model of SM [45], Slobodin et al. in press) that considers a child development within broader family, social, and cultural contexts. At the clinical level, our findings underscore the need for family-based interventions aimed at increasing parents' awareness of their parenting behavior and its impact on their children development. A recent systematic review of the SM interventions for children and adolescents [19] revealed that half of the 25 identified studies involved parents. Research has shown that parenting interventions that teach parents how to maximize opportunities for child verbalization while eliminating accommodation strategies are effective in reducing SM symptoms [42]. For example, Catchpole et al. [9] demonstrated the effectiveness of the combination between parent child interaction and behavioral intervention in improving child speaking in community, school, and home settings. However, most SM interventions studies are limited by small sampling size, insufficient reporting, and lack of power [19]. Further research, especially of high quality, is needed to determine the absolute and relative efficacy of parent–child interventions for SM.

## Data Availability

The data that support the findings of this study are available from the corresponding author upon request.
